# Case of Calculus in the Bladder—Death from Exhaustion

**Published:** 1849-06

**Authors:** W. H. Van Buren


*Case of Calculus in the Bladder—Death from Exhaustion.* By W. H. Van Buren, M. D.—Dr. Van Buren related the particulars of the case of an old gentleman, of 72, who was attacked, five years ago, with retention of urine, of which he had frequent repetitions. He labored under calculus, which gave no trouble and of which no rational signs except frequent micturition existed. Finally, he had an attack of vomiting for 72 hours in succession, which nothing allayed, and he gradually sunk and died from serous effusion into the brain. with diseased heart and exhaustion.

The omentum adhered to the ileum and ascending colon. Recent grumous blood and pus escaped on raising it. The appendix vermiformis was ulcerated at three different points, and attached to the ileum. In the colon, two inches from the valve, was an ulcer three-fourths of an inch in diameter, and a smaller one within it. Old false membrane on the inner surface of the colon. The mucous membrane of the bladder thickened. All the lobes of the prostate immensely enlarged. The peritoneal covering of the bladder very fat. A sacculus behind third lobe. Something very hard felt in third lobe of prostate. The prostate, posterior of the urethra, was not diminished in calibre. The ureter was very much dilated and the inferior infundibula very much enlarged. Pelvis of kidneys not. Substance of the kidneys partially absorbed.—*New York Journal of Medicine.*

